# Association Between Different Biomarkers and Initial Orthodontic Tooth Movement in Children and Adults: A Systematic Review and Meta-Analysis

**DOI:** 10.7759/cureus.78483

**Published:** 2025-02-04

**Authors:** Anisha Rajan CM, Sumeet Ghonmode, Suryakant Powar, Priya Rajput, Pallavi Chaudhary

**Affiliations:** 1 Orthodontics and Dentofacial Orthopaedics, Government Dental College and Hospital, Mumbai, Mumbai, IND

**Keywords:** cytokines, gingival crevicular fluid, interleukins, orthodontics, osteoprotegerin, saliva, tooth movement

## Abstract

The present systematic review aims to bridge the existing knowledge gap by evaluating the influence of various biomarkers on initial orthodontic tooth movement in children and adults. A systematic electronic search was conducted using relevant keywords across the databases PubMed, Google Scholar, and EBSCOhost to identify articles published in English until December 2023. The “Risk of Bias (ROB) in Non-randomized Studies of Interventions” (ROBINS-I) tool was used to assess the risk of bias, and the Grading of Recommendations Assessment, Development and Evaluation (GRADE) approach was applied to critically appraise the quality of evidence. A total of 10 studies were identified, all of which were non-randomised clinical trials that compared biomarker expression in patients belonging to the growing age group (children, juveniles, or adolescents) and adults, using gingival crevicular fluid (GCF) or saliva analysed by the ELISA method. Overall, younger patients consistently exhibited faster and more pronounced biological responses to orthodontic forces, with biomarker levels peaking within the first 72 hours of force application.

## Introduction and background

Orthodontic treatment (OT) primarily involves the movement of teeth by inducing bone remodelling in the preferred direction. Bone remodelling refers to the resorption of the alveolar bone on one side of the socket and formation on the other, allowing the tooth to exhibit bodily movement within the alveolar bone of the jaw. While this is the end product, the process itself involves a complex cascade of cellular interactions down to the molecular level. The success of OT largely depends on these biological responses, which result from the interplay between undifferentiated mesenchymal cells and specialised cells such as fibroblasts, osteoblasts, and osteoclasts [1]. Mechanical forces induce the release of pro-inflammatory cytokines, growth factors, and enzymes that orchestrate bone resorption on the pressure side and bone formation on the tension side. Biomarkers such as receptor activator of nuclear factor kappa-Β ligand (RANKL), osteoprotegerin (OPG), interleukins (IL-1β, IL-6), and prostaglandins (PGE2) play a pivotal role in regulating these processes [1,2]. RANKL actively stimulates osteoclasts to induce bone resorption, whereas OPG acts as a decoy receptor for RANKL, inhibiting its activity. Interleukins and PGE2 are pro-inflammatory cytokines that recruit inflammatory cells, initiating a cascade of bone resorption.

These cells and their biological mediators are abundant in younger individuals, accounting for a rapid bodily response. Thus, OT is preferably performed at a young age when there is still room for the growth of an individual at a favourable pace. However, for several reasons, the OT may get delayed beyond adolescence and the patients may seek it at a later stage during adulthood. These reasons include a lack of awareness or resources, fear of orthodontic treatment, reluctance to comply with long-term treatment, and limited access to healthcare facilities [3,4]. It is still important that treatment is carried out, as patients with malocclusions may face social stigma and humiliation, which can adversely affect their confidence and quality of life. Although not ideal, adult orthodontics remains necessary. Planning OT for adults is challenging due to the diminished remodelling capacity of the bone, reduced physiological response, vascular supply, biological mediators, and undifferentiated cells [5]. Consequently, performing OT in such individuals requires prolonged treatment durations given the slower rate of tooth movement.

Studying biomarkers in the adult population indicated for orthodontic treatment can aid orthodontists in making effective clinical decisions to optimise their treatment plans. Gingival crevicular fluid (GCF) and saliva serve as valuable diagnostic media for monitoring these biomarkers, which have also been studied during OT [6-8]. A recent systematic review identified fluctuations in RANKL, OPG, IL‐1β, and PGE2 at different treatment stages, particularly after orthodontic activation [9]. However, the review concluded with low certainty that orthodontic tooth movement had little to no effect on salivary biomarkers. Despite the critical role of biomarkers in orthodontic tooth movement, a comprehensive understanding of their variations between children and adults remains lacking. While some studies have explored individual biomarkers in isolation, the interplay of multiple biomarkers in age-specific responses is yet to be thoroughly examined. Moreover, the non-invasive nature of GCF and saliva sampling makes them a preferable choice for researchers analysing biomarkers in large population samples.

The present systematic review aims to bridge the existing knowledge gap by evaluating the influence of various biomarkers on initial orthodontic tooth movement in children and adults. The objective of the review is to understand age-related differences in biomarker levels and their impact on orthodontic outcomes. This would aid orthodontists in tailoring optimal treatment plans for patients of different age groups.

## Review

Methodology

Protocol Development

This systematic review was conducted in accordance with the Preferred Reporting Items for Systematic Review and Meta-Analysis (PRISMA) guidelines [10]. The review protocol was registered in the Prospective Registration of Systematic Reviews (PROSPERO) under the identifier CRD42023480594. The review sought to answer the research question: “Is there any association between different biomarkers in GCF and initial orthodontic movements in children and adults?”

Search Strategy

A systematic electronic search was performed across the databases PubMed, Google Scholar, and EBSCOhost to identify relevant articles published in the English language from January 2000 to December 2023. The search was conducted using the following keywords and their combinations: “gingival crevicular fluid” (MeSH term) AND “orthodontic tooth movement” (MeSH term); “orthodontics” (MeSH term) AND “biomarkers” (MeSH term); “prostaglandins” (MeSH term) AND “cytokines” (MeSH term) AND “interleukins” (MeSH term); “children and adults” (MeSH term) AND “saliva” (MeSH term) AND “comparative study” AND “cross-sectional study” AND “prospective study” (MeSH term); “randomized controlled trials” (MeSH term).

To identify grey literature and unpublished clinical trials, Greylist and OpenGrey were used, and cross-referencing of the identified articles was performed. A manual search of orthodontic journals, including the *American Journal of Orthodontics and Dentofacial Orthopedics*, *Angle Orthodontist*, *European Journal of Orthodontics*, *Journal of Orthodontics*, *Journal of Oral Biology and Craniofacial Research*, and *Journal of Craniofacial Research*, was also conducted.

Selection of the Studies

Clinical studies, comparative studies, and prospective studies investigating the association between different biomarkers in GCF or saliva and initial orthodontic tooth movement in children and adults were included in the present systematic review. Only studies with full-text availability in the English language were considered. Reviews, abstracts, letters to the editor, editorials, animal-based studies, and in vitro studies were excluded. Studies with inadequate outcomes relevant to biomarker expression or ambiguity regarding study design were also excluded.

The authors (AR and PR) conducted a two-phase screening and selection of articles. The first phase involved mutual screening of titles and abstracts from the search results. Articles deemed eligible for inclusion were procured and subsequently screened independently by the authors (AR and PR) in the second phase for final inclusion in the review. In case of uncertainty regarding the selection of any study, authors SG and SP were consulted for their expertise. Corresponding authors of the respective articles were contacted via email to obtain any essential permissions or missing data.

Data Extraction

Data related to study samples (size, age, gender), selection criteria used by the authors, treatment methods employed, samples collected, and biomarkers assessed at various time points over a defined follow-up period were extracted independently from the included articles by two groups of authors (AR and SG, PR and SP). Any discrepancies were resolved through discussion and mutual agreement between the two groups.

Assessment of Risk of Bias and Quality of Evidence

Given the nature of the included studies, the “Risk of Bias (ROB) in Non-randomized Studies of Interventions” (ROBINS-I) tool was used to assess the risk of bias [11]. For the critical appraisal of the quality of evidence in each study included in the review, the Grading of Recommendations Assessment, Development and Evaluation (GRADE) approach was adopted [12].

Statistical Analysis

The standardised mean difference with a 95% confidence interval was calculated for continuous outcomes. The significance of any discrepancies in the estimates of the treatment effects across different trials was assessed using the Cochrane Handbook for Systematic Reviews for heterogeneity and the I² statistic [13]. A fixed-effects model (Mantel-Haenszel method) was used if no heterogeneity was observed (p > 0.05 or I² ≤ 24%); otherwise, a random-effects model (DerSimonian-Laird method) was applied [14,15]. All statistical analyses were performed using RevMan 5.3 (Cochrane Collaboration, Software Update, Oxford, UK). The significance level was set at p < 0.05.

Investigation of Publication Bias

To test for the presence of publication bias, the relative symmetry of the individual study estimates was assessed around the overall estimates using Begg’s funnel plot. A funnel plot (plot of effect size versus standard error) was generated. Asymmetry in the funnel plot may indicate publication bias or biases related to sample size; however, it may also reflect a true relationship between trial size and effect size [16].

Results

Study Methods

**Figure 1:**
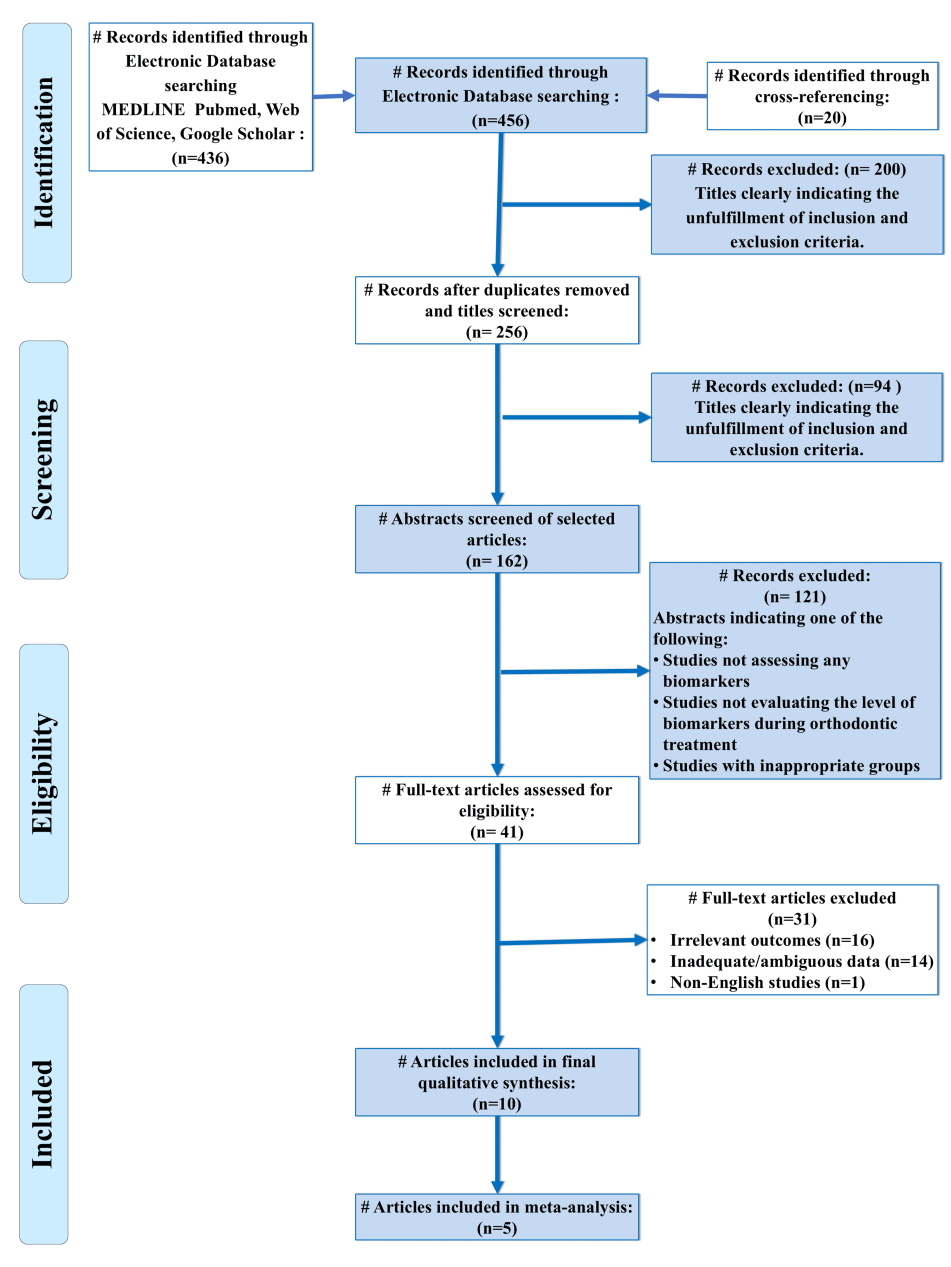
PRISMA flow diagram indicating the selection process of the articles in the present systematic review PRISMA: Preferred Reporting Items for Systematic Review and Meta-Analysis.

The systematic review identified a total of 10 studies that satisfied the selection criteria (Figure 1). The data extracted from the included studies are comprehensively summarised in Table 1 [17-26]. All these studies were non-randomised clinical trials that focused on comparing biomarker expression in patients belonging to the growing age group (children, juveniles, or adolescents) and adults. The levels of these biomarkers were analysed predominantly in GCF (n = 9), with the exception of Mann et al. (2019), who analysed them in the saliva of the subjects [22]. ELISA was the most commonly used method across eight studies, while one study each used enzyme immunoassay (EIA) and a biochemical assay [18,20].

**Table 1:** Data extracted from the studies included in the present systematic review M: male, F: female, C/Ch: children, A: adults, J: juveniles, GCF: gingival crevicular fluid, BPE: basic periodontal examination, ELISA: enzyme-linked immunosorbent assay, EIA: enzyme immunoassay, MBT: McLaughlin, Bennett, Trevisi (bracket system), NiTi: nickel titanium, NS: not specified, OPG: osteoprotegerin, PD: probing depth, PGE2: prostaglandin E2, PTX-3: pentraxin-3, RANKL: receptor activator of nuclear factor kappa-Β ligand, SD: standard deviation, SS: stainless steel, T0: baseline time point, T1: time point 1, TNF-α: tumour necrosis factor-alpha, ↑: increase, ↓: decrease, tx: treatment.

Author(s)	Country	Participants	Age (mean ± SD)	Gender Distribution	Selection Criteria	Intervention	Treatment Method	Samples Collected	Time Points	Biomarkers Studied	Follow-Up Period	Analysis Method	Key Findings	Conclusion	Limitations
Kawasaki et al. (2006) [17]	Japan	30 (15 C, 15 A)	C: 15.1 ± 2.8 years; A: 31 ± 3.6 years	C: 7 M, 8 F; A: 6 M, 9 F	Healthy periodontal tissues (probing depth ≤3 mm); no periodontal bone loss; no antibiotics or anti-inflammatory drugs in recent history	Retraction of upper canines using edgewise brackets and elastomeric chain delivering 250 g initial force	Edgewise brackets (0.018-inch slot); retraction along 0.018-inch archwire	GCF from the distal cervical margins of experimental and control teeth	0, 1, 24, and 168 hours after force application	RANKL, OPG	7 days	ELISA	• Juveniles: 1.23 mm movement; Adults: 0.82 mm. • RANKL ↑, OPG ↓ after 24h. • RANKL/OPG ratio higher in J.	• Tooth movement ↓ with age due to lower RANKL/OPG ratio in GCF.	• Small sample • Response variability.
Chibebe et al. (2009) [18]	Brazil	48 (25 C, 23 A)	C: 13.6 ± 2.1 years; A: 24.1 ± 2.1 years	C: 15 F, 10 M; A: 16 F, 7 M	Good general health; no anti-inflammatory drugs (2 months); no antibiotics (6 months); periodontally healthy; no smoking; required buccal/labial tooth movement as part of orthodontic treatment	Orthodontic brackets (GAC International) with 0.012 Nitinol wire delivering ~0.7 N force	Maxillary right lateral incisors with 0.012 Nitinol wire	GCF from the mesio/buccal gingival crevice of experimental teeth	Baseline (T0), 2 days, 21 days, 28 days	PGE2	28 days	EIA	• J: PGE2 ↑ baseline to 21d, ↓ after 21d. • A: PGE2 stable.	• PGE2 varies by age, linked to faster movement in juveniles.	• 28-day limit • Small sample • No analysis of other mediators.
Surlin et al. (2012) [19]	Romania	29 (16 C, 13 A)	C: 13.81 ± 0.98 years; A: 28.23 ± 3.37 years	C: 9 F, 7 M; A: 8 F, 5 M	Good general health; non-smokers; clinically and radiologically healthy periodontal tissues; no recent antibiotic or anti-inflammatory drug use; good oral hygiene; required upper canine distalisation with first premolar extraction	Application of orthodontic forces to upper canines using fixed appliances	Stainless steel 0.018" Roth brackets, 0.012" NiTi archwire, lacebacks, and transpalatal arch	GCF from test and control canines	-1 h (baseline), 4 h, 8 h, 24 h, 72 h, 1 week, 2 weeks	Pentraxin-3 (PTX-3)	2 weeks	ELISA	• PTX-3 peaked at 24h, returned baseline: 1w adults, 2w young. • Faster response in young.	• PTX-3 key in periodontal remodeling, faster in young.	• 2w follow-up • Small sample • Only 1 biomarker assessed.
Shetty et al. (2015) [20]	India	28 (14 C, 14 A)	C: 11 ± 4 years; A: 25 ± 5 years	NS	Fixed orthodontic therapy with bilateral first premolar extractions; no systemic diseases; no medication in past 3 months; good periodontal health; compliance with oral hygiene	Retraction of maxillary canines using Nickel Titanium coil springs	Fixed appliances with transpalatal arch reinforcement and retraction of canine using 0.018 stainless steel round wire	GCF from the maxillary right canine	Baseline, Day 7, Day 21, Day 42	Acid and alkaline phosphatases	6 weeks	Biochemical assays using semi-automated analyzer	• Alkaline phosphatase higher in children. • Acid phosphatase ↑ at Week 3.	• Faster movement in children linked to higher bone activity markers.	• Small sample • One biomarker • No long-term follow-up.
Vujacic et al. (2016) [21]	Serbia	20 (10 C, 10 A)	C: 13 ± 0.98 years; A: 20 ± 1.6 years	Gender distribution not specified	Good general health; no antibiotics within 3 months; no anti-inflammatory drugs within 1 month; healthy periodontium with 2 mm probing depth	Orthodontic elastic separators placed between the second premolars and first molars	Separator-induced tooth movement	GCF from treatment sites between the second premolar and the first permanent molar	Baseline, 24 h, 72 h, 168 h	IL-1β and IL-6	7 days	ELISA	• IL-1β, IL-6 peaked at 24h, 168h. • IL-6 ↑ more in children. • Tooth movement: C: 1.08 mm; A: 0.89 mm.	• IL-1β, IL-6 critical in early movement; children show faster cytokine responses.	• Small sample • No gender-specific analysis • Short follow-up.
Mann et al. (2019) [22]	India	20 (10 C, 10 A)	C: 12–18 years; A: >18 years	10 M, 10 F	Healthy patients; fixed orthodontic treatment; good oral hygiene; no systemic/periodontal diseases; no tobacco-related habits; no medication history	Fixed orthodontic appliance placement with 0.022-inch slots	Sequential archwire placement	Saliva	T0: Baseline; T1: 1 hour post first wire; T2: 1 month; T3: 1 hour post second wire	IL‑1β, PGE2	1 month	ELISA	• PGE2, IL-1β ↑ at T1, ↓ at T2, ↑ at T3. • Levels higher in adults.	• Salivary IL-1β, PGE2 ↑ during ortho tx. • No pain difference between ages.	• Small sample • No malocclusion severity analysis • Short follow-up.
Afshar et al. (2020) [23]	Iran	20 (10 C, 10 A)	C: 12–19 years; A: 20–33 years	13 F (65%), 7 M (35%)	Satisfactory general health, no antibiotics (6 months prior), no anti-inflammatory medication (1 month prior), no periodontal disease (PD ≤ 3 mm, no attachment loss), Class I malocclusion, incisor irregularity index 3–6 mm, permanent dentition, no tooth removal in the treatment plan	Fixed orthodontic treatment with MBT 0.022 slot bracket system	Bonding and insertion of 0.014-inch NiTi archwire followed by sample collection	GCF from maxillary right central incisor (disto-labial aspect)	Baseline (before bonding), 24 hours, 7 days, 28 days after bonding	TNF-α and IL-1β	28 days	ELISA	• TNF-α, IL-1β ↑ post-tx, peak 24h. • No significant age or gender differences.	• TNF-α, IL-1β levels ↑ during early ortho tx.	• Small sample • Uniform protocols • No analysis of other cytokines.
Chelărescu et al. (2021) [24]	Romania	20 (8 C, 12 A)	C: 11–16 years; A: 17–28 years	11 F (55%), 9 M (45%)	Indication for orthodontic treatment with premolar extraction, periodontal health (BPE score 0, no radiographic bone loss), no systemic diseases or medications	Fixed orthodontic treatment using MBT technique with metallic brackets and closed coil springs	Brackets bonded using Transbond XT and forces applied with 0.19 × 0.25 SS wires	GCF from distal gingival sulcus of maxillary canines	Baseline (T0), 24 hours after activation of orthodontic forces (T1)	IL1β and IL6	24 hours	ELISA	• IL1β ↑ T0 to T1, higher in adolescents (42.96 pg/μL vs. 17.93 pg/μL). • IL6 detectable in 1/3 sites.	• IL1β ↑ faster in adolescents, correlating with faster movement.	• 2 time points • Small sample • Low IL6 detection.
Afshar et al. (2021) [25]	Iran	20 (10 C, 10 A)	19.11 ± 6.23 years	14 F (70%), 6 M (30%)	Good general health, no recent antibiotics or anti-inflammatory use, absence of periodontal disease, Class I malocclusion, incisor irregularity index of 3–6 mm, permanent dentition, no extractions needed in treatment	Fixed orthodontic treatment with MBT 0.022-inch brackets and initial 14 NiTi archwire	GCF collected with paper strips from the gingival sulcus of maxillary central incisors	GCF from distolabial site	Baseline, 24 hours, 7 days, and 28 days after bonding	IL-8	28 days	ELISA	• IL-8 ↓ day 1, ↑ later but not to baseline. • No significant age or gender differences.	• IL-8 ↓ early, later ↑. • No significant age/gender differences.	• Small sample • Narrow age range • Short follow-up.
Kumar et al. (2022) [26]	India	20 (10 C, 10 A)	C: 11-16 years, A: 17-28 years	10 M, 10 F	Good periodontal health, no systemic diseases, no recent antibiotics or anti-inflammatory drugs, premolar extraction	fixed orthodontic treatment using 0.022 inch MBT brackets and 0.19 × 0.25 stainless steel wires and canine retraction using nickel-titanium closed coil springs	GCF collected using Periotron 8000 from the distal zone of canines	GCF from the distal zone of the canine	Baseline (T0), 24 hours after treatment (T1)	IL-1β, IL-6	24 hours	ELISA	• IL-1β ↑ baseline to 24h in both groups. • IL-6 detectable in 1/3 samples. • Adolescents: higher IL-1β vs YA.	• Adolescents show faster tissue response and higher IL-1β expression.	• Two time points (T0, T1) • Small sample • IL-6 undetectable in most cases.

Study Samples

The sample size across the studies ranged from 20 to 48, with a total of 255 subjects included in the present systematic review. Six out of the 10 studies had a sample size of 20 subjects. An almost equal number of children and adults were considered across all studies (128 children and 127 adults). The age of children ranged from 11 to 19 years, while that of adults was less than 28 years, with the exception of Kawasaki et al. (2006), who reported a mean age of 31 ± 3.6 years for the adult population [17]. The studies were conducted on systemically healthy individuals with good periodontal health.

Orthodontic Interventions

Regarding the orthodontic aspect, alignment and levelling were carried out with nickel-titanium archwires in five studies, while canine retraction with nickel-titanium closed-coil springs was performed in three studies. Retraction of upper canines with elastomeric chains and tooth movement induced after placing separators were assessed in one study each, respectively. The follow-up period across the studies varied from 24 hours to one month.

The various biomarkers analysed included cytokines or pro-inflammatory mediators such as interleukins (IL-1β, IL-6, IL-8), tumour necrosis factor-alpha (TNF-α), prostaglandin E2 (PGE2), and those related to bone remodelling, including receptor activator of nuclear factor kappa-Β ligand (RANKL), osteoprotegerin (OPG), pentraxin-3 (PTX-3), alkaline phosphatase, and acid phosphatase. Among the biomarkers studied, IL-1β was the most frequently analysed (n = 5 studies), followed by IL-6 (n = 3 studies) and PGE2 (n = 2 studies); the remainder were analysed in one study each, respectively.

Across multiple studies, younger patients (juveniles and adolescents) consistently demonstrated faster and more pronounced biological responses to orthodontic forces compared to adults. A higher expression of RANKL, OPG, alkaline phosphatase, acid phosphatase, IL-1β, and IL-6 was observed among individuals in the growing age group compared to adults [17,20,21,24]. Afshar et al. (2020, 2021) and Kumar et al. (2022) reported no significant differences in TNF-α, IL-1β, or IL-8 levels between males and females [23,26]. Kawasaki et al. (2006) and Vujacic et al. (2016) identified peaks in RANKL, IL-1β, and IL-6 at 24 to 168 hours post-force application [17,21]. Chelărescu et al. (2021) noted a significant increase in IL-1β at 24 hours in adolescents [24].

Temporal Trends in Biomarker Expression

Most studies observed a peak in biomarker levels shortly after the application of orthodontic force, followed by a gradual decline, particularly in adolescents. These trends align with the acute inflammatory response phase, characterised by the rapid recruitment of inflammatory mediators and bone remodelling markers.

Bone Remodelling and Orthodontic Perspective

The biomarkers studied, including RANKL, OPG, IL-1β, IL-6, PGE2, and alkaline phosphatase, are central to bone remodelling during orthodontic tooth movement. Kawasaki et al. (2006) emphasised the pivotal role of the RANKL/OPG axis in mediating osteoclast activity, with younger patients exhibiting faster bone resorption and deposition cycles [17]. Shetty et al. (2015) further corroborated this with elevated phosphatase levels in children, indicative of heightened bone turnover [20].

In the context of aseptic inflammation and tissue remodelling, Surlin et al. (2012) highlighted PTX-3 as a potential marker for monitoring early orthodontic responses [19]. Vujacic et al. (2016) and Mann et al. (2019) reinforced the importance of IL-1β and PGE2 in driving the inflammatory cascade and subsequent tooth movement [21,22].

Age- and Gender-Related Differences in Biomarker Expression

Kawasaki et al. (2006) reported higher levels of RANKL and lower OPG levels in juveniles, along with a significantly higher RANKL/OPG ratio [17]. Chibebe et al. (2010) found that juveniles exhibited greater fluctuations in PGE2 levels over the study period, peaking at 21 days, whereas adults showed stable levels [18]. These fluctuations may explain the faster tooth movement observed in younger patients. Vujačić et al. (2016) demonstrated significantly higher IL-1β and IL-6 levels in children compared to adults during the acute phase of orthodontic tooth movement, correlating with greater cytokine-mediated inflammation in younger participants [21]. Maan et al. (2019) observed elevated salivary IL-1β and PGE2 levels in adults compared to children during early orthodontic treatment [22]. However, pain perception did not significantly differ between the groups. No significant differences in biomarker expression were observed based on gender across the studies [23,25].

Time-Dependent Changes in Biomarkers

Studies consistently reported peak biomarker levels at 24-72 hours after orthodontic force application, followed by a gradual decline. Karimi-Afshar et al. (2021) observed a significant decrease in IL-8 levels on the first day post-treatment, followed by an increase over time, though baseline levels were not reached within the 28-day period [25]. Chelărescu et al. (2021) reported a significant increase in IL-1β at 24 hours for both adolescents and young adults, with the former demonstrating a greater response [24].

Biological Markers of Bone Remodelling

Surlin et al. (2012) highlighted the role of pentraxin-3 (PTX-3) in aseptic inflammation and periodontal remodelling. PTX-3 levels peaked earlier in younger participants, suggesting faster biological responses in juveniles compared to adults. Shetty et al. (2015) noted consistently higher levels of alkaline and acid phosphatases in children, reflecting heightened osteoblastic and osteoclastic activity during orthodontic tooth movement.

Overall Trends

Younger patients consistently exhibited faster and more pronounced biological responses to orthodontic forces, likely due to higher metabolic activity and cytokine expression in periodontal tissues. Biomarker levels peaked within the first 72 hours of orthodontic force application, suggesting this period is critical for assessing biological activity and optimising treatment interventions. While non-invasive sample collection methods (e.g., GCF and saliva) were effective, the variability in biomarker detection thresholds and analysis techniques warrants caution in interpreting findings.

Risk of Bias

**Figure 2:**
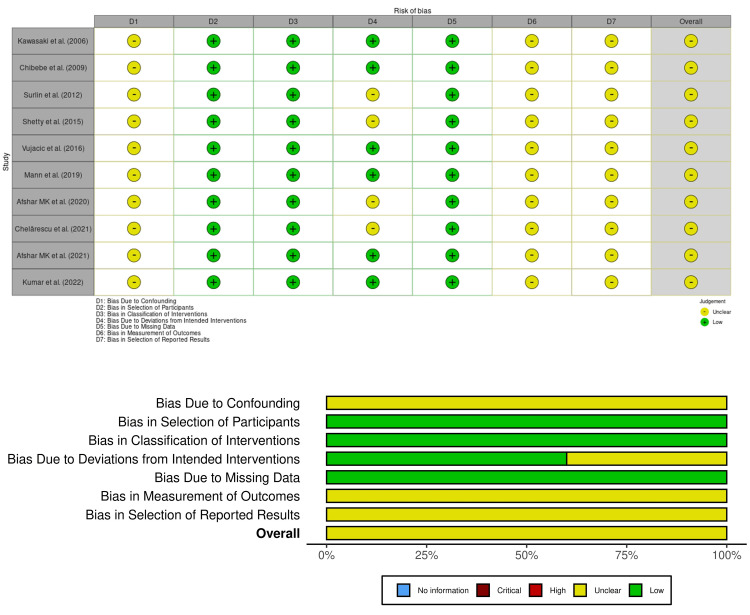
Risk of bias across studies in the systematic review, individual (upper plot) and summary (lower plot) Data taken from refs. [17-26].

The risk of bias assessment using the ROBINS-I tool indicated that most studies were classified as having a moderate risk of bias (Figure 2). For instance, studies by Kawasaki et al. (2006) and Chibebe et al. (2010) lacked randomisation, blinding, and allocation concealment, introducing potential systematic bias [17,18]. Similarly, the study by Surlin et al. (2012) had issues with baseline differences and a limited sample size, which may have confounded the results [19]. The studies by Vujačić et al. (2016) and Karimi-Afshar et al. (2020) also demonstrated moderate bias due to their non-randomised design and incomplete reporting of strategies to minimise confounding [21,23]. Despite these limitations, all studies were methodologically adequate for observational research, providing detailed intervention and outcome measures.

GRADE Assessment

Using the GRADE approach, the quality of evidence across the studies ranged from low to moderate (Table 2). Studies such as Kawasaki et al. (2006) and Surlin et al. (2012) were downgraded for imprecision due to their small sample sizes and wide confidence intervals, which limited the certainty of their findings [17,19]. Inconsistency was noted in the results of studies such as Chibebe et al. (2010) and Maan et al. (2019), where differing trends in biomarker levels were reported at similar time points [18,22]. Indirectness was low overall, as the studies directly addressed the review question regarding cytokine levels during orthodontic tooth movement. The likelihood of publication bias appeared low, given the lack of evidence for selective reporting in the included studies.

**Table 2:** Appraisal of quality of evidence by GRADE approach GRADE: Grading of Recommendations Assessment, Development and Evaluation.

Author(s)	Risk of Bias	Inconsistency	Indirectness	Imprecision	Publication Bias	Overall Quality
Kawasaki et al. (2006) [17]	Moderate	Low	Low	Moderate	Low	Moderate
Chibebe et al. (2009) [18]	Moderate	Low	Low	Moderate	Low	Moderate
Surlin et al. (2012) [19]	Moderate	Moderate	Low	High	Low	Low
Shetty et al. (2015) [20]	Moderate	Low	Low	Moderate	Low	Moderate
Vujacic et al. (2016) [21]	Moderate	Moderate	Low	High	Low	Low
Mann et al. (2019) [22]	Moderate	Low	Low	Moderate	Low	Moderate
Afshar et al. (2020) [23]	Moderate	Moderate	Low	Moderate	Low	Moderate
Chelărescu et al. (2021) [24]	Moderate	Moderate	Low	High	Low	Low
Afshar et al. (2021) [25]	Moderate	Low	Low	Moderate	Low	Moderate
Kumar et al. (2022) [26]	Moderate	Low	Low	Moderate	Low	Moderate

Synthesis of Results

The meta-analysis was conducted to evaluate the effect of biomarkers on the rate of initial orthodontic tooth movement between children and adults. Five studies containing data on 116 teeth subjected to initial orthodontic tooth movement under various biomarkers were included, with n = 58 teeth evaluated in the adult group and n = 58 teeth in the children/juvenile group for assessing the rate of initial orthodontic tooth movement [17,19,21,25,26].

**Figure 3:**
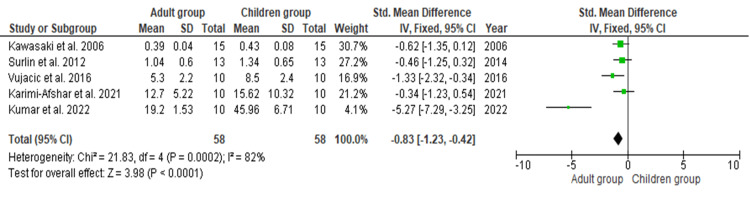
Rate of initial orthodontic tooth movement between adults and children SD: standard deviation; CI: confidence Interval. Data taken from refs. [17-26].

As shown in Figure 3, the SMD is -1.26 (-2.26 to -0.25), and the pooled estimates favour adults, signifying that the overall rate of initial orthodontic tooth movement under the influence of biomarkers is, on average, 1.26 times greater in children. This difference is statistically significant (p < 0.05).

Publication Bias

**Figure 4:**
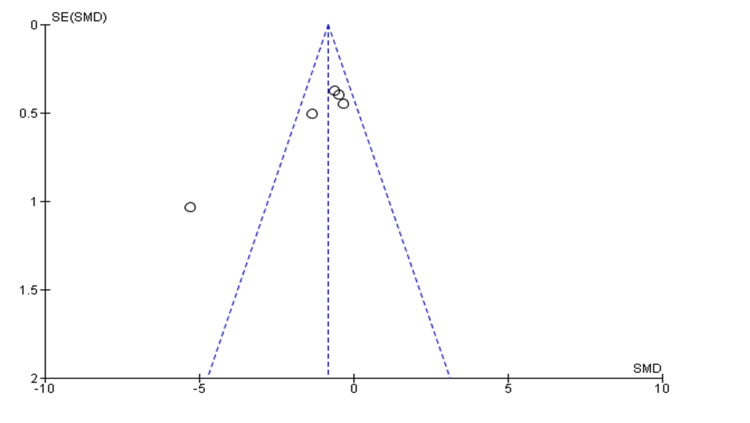
Begg’s funnel plot with 95% confidence intervals indicating an absence of publication bias Data taken from refs. [17-26].

The funnel plot showed significant asymmetry, indicating an absence of publication bias, as shown in Figure 4.

Discussion

The biological processes underlying orthodontic tooth movement are complex, involving a complex interplay of biomolecular alterations. The findings of the present systematic review revealed the role of cytokines and mediators of bone remodelling during orthodontic tooth movement. Orthodontic forces create a microenvironment characterised by mechanical stress, which translates into biological signals. These signals activate cells within the periodontal ligament (PDL) and alveolar bone, initiating bone resorption on the pressure side and bone formation on the tension side. The markers investigated in the included studies were IL-1β, IL-6, IL-8, RANKL, OPG, PGE2, and PTX-3, which play a pivotal role in orchestrating these processes.

The cytokines IL-1β and IL-6 are among the first mediators released in response to mechanical loading of teeth [27]. IL-1β is a key pro-inflammatory cytokine responsible for upregulating osteoclast differentiation and activity through the RANKL pathway and was thus analysed in most of the studies [28]. Increased levels of IL-1β in the studies included in the present review correlate with enhanced bone resorption on the pressure side, facilitating tooth movement [21,26]. The findings that adolescents exhibit higher IL-1β levels than adults align with known age-related differences in cellular activity. Younger patients typically have a more robust inflammatory response due to greater PDL vascularisation, higher metabolic rates, and an abundance of progenitor cells capable of osteoclastogenesis [29].

IL-6 serves as both a pro-inflammatory mediator and a regulator of bone metabolism. It promotes osteoclast differentiation indirectly through the induction of RANKL expression in osteoblasts [30]. The transient elevation of IL-6 in GCF, as reported across the studies included in the present review, highlights its role in the early phase of tooth movement. However, its lower detectability in certain samples may reflect methodological limitations or variations in sample collection timing [24]. Likewise, IL-8 is an equally essential cytokine that functions as a chemoattractant for neutrophils and other immune cells. Its decrease during the first day of treatment may indicate the resolution of initial vascular changes and a shift towards sustained bone remodelling processes [25]. The subsequent increase in IL-8 levels at later time points suggests its role in recruiting cells involved in tissue repair and remodelling.

PGE2 is known for its role in amplifying inflammatory responses and enhancing osteoclastogenesis. The significant fluctuations in PGE2 levels in juveniles, as observed by Chibebe et al. (2010), further support the heightened inflammatory response in younger individuals [18]. The early recruitment and activation of osteoclast precursors correspond to the active phases of tooth movement.

The RANKL/OPG axis is central to bone remodelling. RANKL binds to its receptor on osteoclast precursors, promoting their differentiation and activation. OPG acts as a decoy receptor, inhibiting RANKL-RANK interactions and thereby regulating osteoclast activity. The findings by Kawasaki et al. (2006) that juveniles exhibit higher RANKL/OPG ratios than adults provide a mechanistic explanation for faster tooth movement in younger patients [17]. This age-related difference can be attributed to a more active bone remodelling environment in adolescents, supported by a higher RANKL availability relative to OPG [3,5,29].

PTX-3 is an acute-phase protein implicated in the regulation of aseptic inflammation and tissue remodelling [31]. Elevated PTX-3 levels observed within 24 hours of orthodontic movement initiation reflect its role in modulating the early inflammatory response [19]. The faster normalisation of PTX-3 levels in adults suggests a more restrained inflammatory milieu, which is consistent with age-related reductions in metabolic activity and cellular turnover.

Adolescents invariably exhibit more dynamic changes in biomarker levels compared to adults across the studies included in the present systematic review. This highlights the biological basis for age-related differences in orthodontic outcomes. Adolescents possess a more vascularised and cellular-rich PDL, which facilitates a rapid release of cytokines and recruitment of osteoclasts [29]. In contrast, adults experience a slower response due to reduced progenitor cell populations, decreased vascularity, and a denser alveolar bone structure [3,5].

These differences have direct clinical implications. Faster tooth movement in adolescents may necessitate adjustments in force magnitude and timing to avoid undesired side effects such as root resorption. Conversely, in adults, prolonged treatment durations may require adjunctive therapies, such as pharmacological modulation of bone remodelling or surgical acceleration techniques, to optimise outcomes.

While the reviewed studies provide valuable insights, several limitations should be addressed. Variations in study design, follow-up durations, and biomarker quantification methods hinder cross-study comparisons. The lack of randomised controlled trials further limits the strength of evidence. Future research should focus on standardised protocols for GCF collection, larger sample sizes, and extended follow-up periods. Additionally, exploring systemic factors such as hormonal influences and genetic predispositions will enhance our understanding of individual variability in biomarker expression and treatment outcomes.

Overall, the dynamic interplay of cytokines and bone remodelling mediators elucidates the biological mechanisms underlying orthodontic tooth movement. Age-related differences in biomarker expression highlight the need for personalised approaches to treatment planning and force application. By integrating biomarker-based monitoring into clinical practice, orthodontists can optimise treatment efficiency while minimising risks, paving the way for biologically driven orthodontic care.

## Conclusions

Within their limitations, the findings of the present systematic review highlight the critical role of biomarkers such as IL-1β, IL-6, IL-8, RANKL, OPG, PGE2, and PTX-3 in mediating the biological processes of orthodontic tooth movement through their regulation of inflammation and bone remodelling. The findings consistently demonstrate age-related differences, with adolescents exhibiting heightened and more dynamic biomarker responses compared to adults, correlating with faster tooth movement and greater osteoclast activity. These differences emphasise the importance of individualised treatment approaches based on patient-specific biological profiles. While the evidence provides valuable insights into the molecular mechanisms of orthodontic therapy, the overall quality is constrained by methodological heterogeneity, limited sample sizes, and the absence of randomised controlled trials. Future studies with standardised protocols and extended follow-ups are warranted to strengthen the evidence base and further refine biomarker-guided orthodontic interventions.
